# Measurement of oxygen transfer from air into organic solvents

**DOI:** 10.1002/jctb.4862

**Published:** 2015-12-29

**Authors:** Hemalata Ramesh, Torsten Mayr, Mathias Hobisch, Sergey Borisov, Ingo Klimant, Ulrich Krühne, John M Woodley

**Affiliations:** ^1^ Department of Chemical and Biochemical EngineeringTechnical University of DenmarkDK‐2800 KgsLyngbyDenmark; ^2^ Institute of Analytical Chemistry and Food ChemistryGraz University of Technology8010GrazAustria

**Keywords:** optical sensor; organic solvents; oxygen transfer

## Abstract

**BACKGROUND:**

The use of non‐aqueous organic media is becoming increasingly important in many biotechnological applications in order to achieve process intensification. Such media can be used, for example, to directly extract poorly water‐soluble toxic products from fermentations. Likewise many biological reactions require the supply of oxygen, most normally from air. However, reliable online measurements of oxygen concentration in organic solvents (and hence oxygen transfer rates from air to the solvent) has to date proven impossible due to limitations in the current analytical methods.

**RESULTS:**

For the first time, online oxygen measurements in non‐aqueous media using a novel optical sensor are demonstrated. The sensor was used to measure oxygen concentration in various organic solvents including toluene, THF, isooctane, DMF, heptane and hexane (which have all been shown suitable for several biological applications). Subsequently, the oxygen transfer rates from air into these organic solvents were measured.

**CONCLUSION:**

The measurement of oxygen transfer rates from air into organic solvents using the dynamic method was established using the solvent resistant optical sensor. The feasibility of online oxygen measurements in organic solvents has also been demonstrated, paving the way for new opportunities in process control. © 2015 The Authors. *Journal of Chemical Technology & Biotechnology* published by JohnWiley & Sons Ltd on behalf of Society of Chemical Industry.

**NOTATION**
K_L_a: oxygen mass transfer coefficient
[O_2_]*: saturation concentration of oxygen
[O_2_]: concentration of oxygen at a given time
pO_2_*: saturation partial pressure of oxygen
pO_2_: partial pressure of oxygen at a given time
H: Henry's Law constant

## INTRODUCTION

Oxygen transfer is important in many bioprocesses employed at industrial scale. In aerobic fermentation and biocatalysis using growing cells, oxygen is used both for cell growth and maintenance. Likewise in biocatalysis, using resting cells or isolated enzymes for chemical synthesis, oxygen is used as a substrate in many reactions such as those catalysed by oxidases^1^ or oxygenases.^2^, ^3^, ^4^, ^5^ In all these biotechnological examples, the measurement of oxygen concentration in solution is essential, in order to provide valuable data which can subsequently be used for process monitoring and/or control.^6^

While most bioprocesses are carried out in an aqueous solution, there is increasing interest in the use of non‐aqueous media to partially, or completely, replace water.^7^ This is motivated in large part by the significant benefits in process intensification that can result from the introduction of poorly water‐soluble organic solvents above their water saturation concentration, resulting in a biphasic system.^8^, ^9^, ^10^, ^11^, ^12^ One particularly interesting consequence of the introduction of an organic solvent is usually better oxygen transfer. Indeed it has even been suggested to use organic solvents as ‘oxygen vectors’ in order to improve the oxygen supply in some fermentation processes.^13^, ^14^ Oxygen transfer is improved due to the far higher solubility of oxygen in many organic solvents compared with water,^15^, ^16^ where the solubility is limited to 5 mL O_2_ L^−1^ water (0% salinity, 30 °C).^17^ Oxygen is usually supplied in air, meaning a maximum oxygen concentration in solution of about 250 µmol L^−1^ oxygen. The higher oxygen solubility in organic solvents gives a higher concentration driving force for mass transfer, resulting in improved transfer rates. On this premise we reasoned that the application of a sensor suitable for measurement of oxygen concentration in organic solvents would be useful.

Indeed, historically the determination of oxygen solubility in organic solvents has proven challenging. Methods reported include titration, use of manometers, photochemical methods, electron spin resonance and chromatography.^18^ However, none of these methods enable continuous measurement. For such an application, Clark‐type electrochemical sensors have been established for the measurement of oxygen concentration in bioprocesses.^19^, ^20^, ^21^ However, despite their long history only a few studies report the use of Clark electrodes in apolar organic solvents^22^ or alcoholic solutions. This is because the membrane used in a Clark electrode is typically made of a polymer which is prone to swelling when exposed to organic solvents, leading to erroneous measurements. More recently, the application of optical oxygen sensors has been reported as an alternative,^23^ using measurement principles based on luminescent quenching of an indicator dye by the molecular oxygen. The indicator dye is embedded in a host polymer composed of sol–gel, silicone and polystyrene. Nevertheless, most of the established optical sensors are not adequate for application in organic solvents either, due to the swelling or dissolution of the polymeric material. More recently some optical sensors with resistance to organic solvents were reported.^24^, ^25^, ^26^, ^27^, ^28^ On this basis we sought to use these sensors for continuous online measurement of oxygen concentration in organic solvents as the basis for the determination of oxygen transfer rates. This is we believe the first report of such measurements.

## EXPERIMENTAL

All solvents, unless specified, were purchased in analytical grade from Sigma Aldrich (Steinhiem, Germany) and used as purchased. The solvent‐resistant oxygen sensors (model OXSOLV) and the FirestingO2 were obtained from PyroScience (Aachen, Germany).

### Sensor characterization in organic solvents

Gas mixtures of defined oxygen partial pressure (*pO_2_*) were obtained using two mass flow controllers (Read‐Ysmart series) from Vögtlin instruments (www.voegtlin.com). Compressed air and pure nitrogen were used as calibration gases. The mixtures were controlled with an oxygen microsensor (model OXR230‐CL4**)** from PyroScience. The calibration gas was passed through a glass trap and subsequently a glass measurement cell (maintained at 25 °C in a water bath). In turn the glass trap and the measurement cell were filled with the organic solvents to be evaluated.

### Reactor

Experiments were carried out in a 250 mL stirred tank reactor equipped with two Rushton turbines (six blades, diameter 2.4 cm, width of the blade was 0.6 cm and height 0.5 cm) on a single drive shaft, and two baffles (of 1 cm width). Both the height of the impellers and the agitation speed were adjustable.

### Calibration of the oxygen sensor

A 2‐point calibration was made for the oxygen sensors by exposing them to air (for 100% DO) and 1% sodium sulphite (for 0% DO). The calibration for the zero reading was carried out with sodium sulphite since it was a fast and an easy‐to‐use calibration method.

### K_L_a measurements for oxygen transfer from air to organic phase

*k_L_a* measurements were made for oxygen transferring from air to heptane, MTBE, isooctane and cyclohexane respectively, using the well‐established dynamic gassing out method.^29^ Nitrogen was used for gassing out and head‐space aeration was employed as a means of oxygen supply.

50 mL of a given organic solvent was placed in the stirred tank reactor (maintained at 37 °C in a water bath) and the stirring speed was set to 80 rpm for all solvents ensuring that the solvent was mixed without disturbing the surface. The low stirring speed ensured that there was no splashing and thus a fixed gas–liquid interfacial area was made available for oxygen transfer. The headspace was flushed with nitrogen until the oxygen concentration in the aqueous phase stabilized (zero reading). Subsequently, the nitrogen flow was stopped and the reactor left open to enable to fill the headspace. Oxygen then transferred from the headspace to a given organic solvent until the two were equilibrated. The increase in oxygen concentration in the respective organic solvent was measured using two oxygen sensors (one attached to each baffle) enabling duplicate measurements. For calculation of the specific interfacial area (a) available for oxygen transfer, the volume of the solvents was considered to be 50 mL. Exceptionally in the case of MTBE, the volume used was 40 mL on account of evaporation losses during degassing, resulting in a higher specific interfacial area.

## RESULTS AND DISCUSSION

In the following section, we report the measurement of oxygen concentration and transfer rate of oxygen from air to neat organic solvents. To that end, the oxygen sensor was first characterized in different solvents followed by use them in organic solvents with the aim of measuring oxygen transfer rates.

### Characterization of oxygen sensor in organic solvents

**Figure 1:**
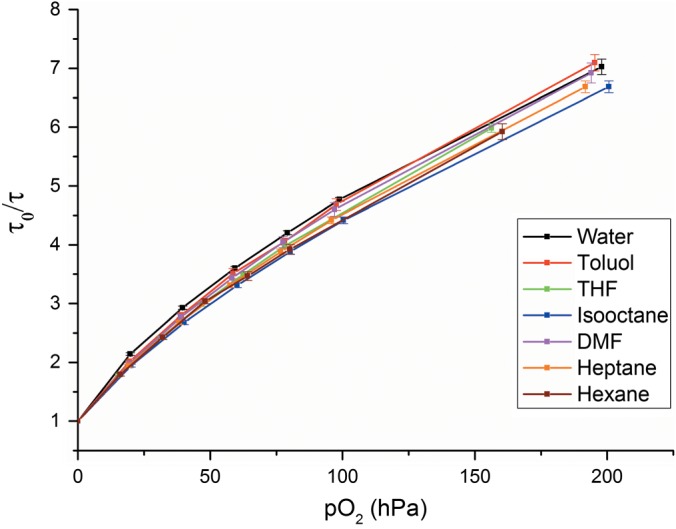
Calibration curves of oxygen sensors in different organic solvents at 25 °C.

Initially, the oxygen sensor was tested in representative organic solvents, covering a wide range of different polarities. The active sensor coating is composed of a Teflon‐like hydrophobic polymer, which makes it resistant to a range of non‐aqueous solvents. The sensor was exposed to the organic solvents for 1 h. The sensors showed a response time (t_90_) of 4 s, fast enough for the measurements required in the dynamic method. Figure 1 shows the calibration curves of the sensor at varying oxygen partial pressures, when exposed to the different organic solvents. The calibration curves are nearly identical for all tested solvents, confirming that the individual solvents do not influence the sensor material.

### Measurement of oxygen transfer rate from gas to organic solvent

The sensors were then used to measure oxygen transfer rates from air to neat organic solvents. The selection of organic solvents was limited to those applicable in biological processes such as fermentation and biocatalysis. The solvents were selected therefore on the basis of a log P > 4 (30) and which also met environmental guidelines, using the ACS GCI solvent selection guide.^9^

**Figure 2:**
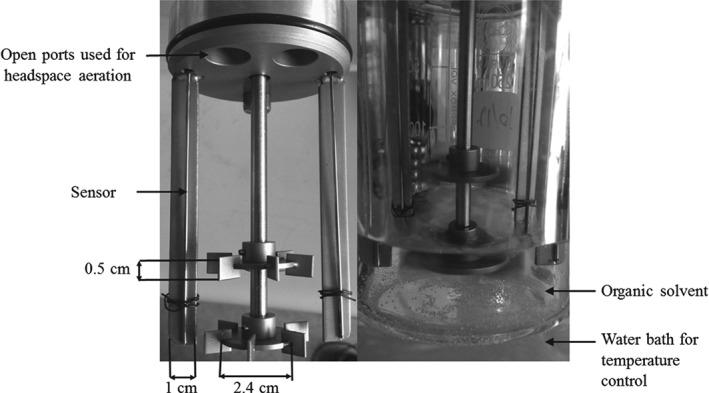
Photograph of the experimental set up. The dimensions of the rotors and baffles are represented on the left and the assembled reactor on the right.

Oxygen transfer rates from the headspace to the organic phase were measured by the well‐established dynamic method.^29^ First, the oxygen concentration in the organic solvent was depleted by flushing the headspace with nitrogen (schematically illustrated in Fig. 2). After flushing with nitrogen the open vessel filled with air, and the subsequent oxygen saturation profile was also obtained through headspace aeration. Bubbled aeration was avoided since it would lead to solvent evaporation, complicating interpretation of the results.

**Figure 3:**
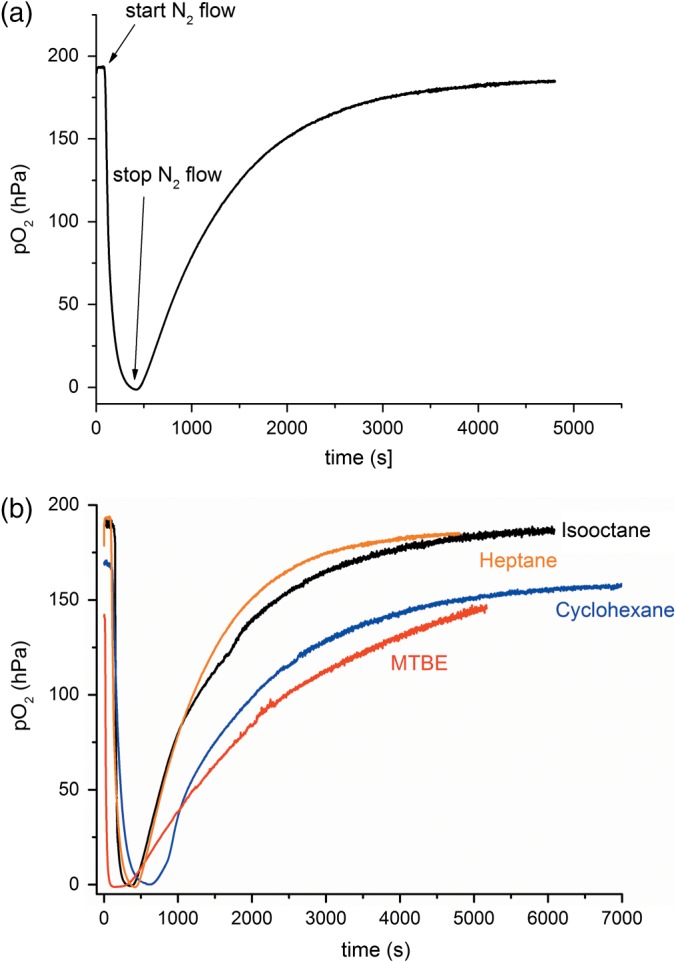
(a) Dynamic method for determination of oxygen transfer rates from the headspace to heptane system. (b) Dynamic method for oxygen transfer measurements from isooctane, cyclohexane, MTBE and heptane.

When the nitrogen sweeps out the air from the headspace, the oxygen concentration becomes zero. When headspace aeration follows, the oxygen concentration rises in the system until it reaches the saturation concentration (as seen in Fig. 3). It is important that the sensors used for *k_L_a* measurements have a fast response in order to avoid any errors due to artefacts as a result of the measurement itself.^30^

The oxygen transfer rate can be described by the equation:

(1) $$\frac{d\left[O_{2}\right]}{dt}=k_{L}a*\left({\left[O_{2}\right]}^{*}-\left[O_{2}\right]\right)-r_{\text{uptake}}$$

where d[*O*_2_]/d*t* represents the rate of change of oxygen concentration, *t* time, [*O*_2_]^*^ and [*O*_2_]_,_ the saturation concentration of oxygen and the concentration of oxygen in the liquid phase at a given time, respectively. *r_uptake_* represents the rate of oxygen uptake in the system. Since there is no active consumption of oxygen, the oxygen uptake rate is zero and the equation can be reduced to:

(2) $$\frac{d\left[O_{2}\right]}{dt}=k_{L}a*\left({\left[O_{2}\right]}^{*}-\left[O_{2}\right]\right)$$

The volumetric mass transfer coefficient is represented by *k_L_a* which is itself composed of *k_L_* representing the mass transfer coefficient and *a* the specific interfacial area.^31^

According to Henry's law, the concentration of oxygen in solution in equilibrium with the gas, [*O*_2_]^*^, is proportional to the partial pressure of oxygen in the gas (pO_2_^*^):

(3) $${\left[O_{2}\right]}^{*}=H*pO_{2}^{*}$$

Re‐writing Equation (2) in terms of partial pressure (*pO*_2_) and saturation partial pressure (*pO*_2_^*^) gives:

(4) $$\frac{HpO_{2}}{dt}=k_{L}a*\left(HpO_{2}^{*}-HpO_{2}\right)$$

Solving Equation (4) gives:

(5) $$\ln\left(pO_{2}^{*}-pO_{2}\right)=-k_{L}a*t+\ln pO_{2\;}^{*}$$

In this way, the *k_L_a* for each solvent can be obtained from the plot of ln (*pO*_2_^*^–*pO*_2_) as a function of time. Partial pressures were measured using the sensors (Fig. 3), *pO*_2_* is the partial pressure recorded at equilibrium and *pO*_2_, the partial pressure recorded at a given time.

*k_L_* values were calculated from the estimated *k_L_a* for the different solvents and are presented in Table 1. As a control water was also tested and from Table 1, it can be seen that the mole fraction of oxygen in the water phase is lower by an order of magnitude than in any of the organic solvents reflecting the higher oxygen solubility measured in the organic solvents.^16^ As expected solubility of oxygen in the solvents reflects the *P_sat_* ^*^ values. The results indicate not only that the sensor can be used to measure oxygen in organic solvent, but can also be used online in a dynamic method, thus facilitating the collection of oxygen transfer data. To the best of our knowledge this is the first time such results have been reported. Such online measurements can be particularly useful when operating a biological system in a Lewis cell^32^, ^33^, ^34^ to measure the mass transfer that occurs between two immiscible liquid phases with a defined interfacial area. The *k_L_* values obtained from the Lewis cell experiments can be used to predict the oxygen transfer rates for a given phase ratio for a well‐mixed large‐scale system. For example, if an oxygen transfer rate from organic solvent to aqueous phase (where the oxygen requiring biocatalytic process occurs) is required, the solvent sensors could be placed in each of the phases (aqueous and organic) and oxygen concentrations measured over time. The result obtained would in turn indicate the mass transfer rates from gas to organic phase and subsequently organic phase to aqueous phase.

**Table 1:** k_L_a and k_L_ values for different solvents at 30°C

Solvent	*k_L_a* (h^−1^)	*k_L_* (m h^−1^)	*P* ^*^_sat_ (mbar)	Mol fraction of oxygen*1000^a^
MTBE	1.73	0.021^b^	150	‐
Cyclohexane	2.56	0.039	160	1.24
Heptane	3.96	0.060	185	2.11
Isooctane	2.88	0.043	189	2.63
Water	7.2	0.108	220	0.02

a As reported in Ref. 16.

b The specific surface interfacial area used for MTBE is calculated with the volume of the liquid 40 mL (the reduced volume considered here is to account for evaporation of solvent during the deoxygenation phase).

## CONCLUDING REMARKS

Oxygen transfer rates from air to neat organic solvents were determined with a solvent‐resistant optical sensor. The study demonstrates for the first time the feasibility of online measurements of oxygen in organic solvents, which was previously limited by unstable sensors. The sensor therefore presents new possibilities in process control of intensified fermentation and biocatalytic processes where changes in oxygen concentrations occur in the presence of organic solvents.

## CONFLICTS OF INTEREST

The authors declare that there are no conflicts of interest.
